# Volatile Flavor of *Tricholoma matsutake* from the Different Regions of China by Using GC×GC-TOF MS

**DOI:** 10.3390/foods14101824

**Published:** 2025-05-21

**Authors:** Yunli Feng, Shaoxiong Liu, Yuan Fang, Jianying Li, Ming Ma, Zhenfu Yang, Lue Shang, Xiang Guo, Rong Hua, Dafeng Sun

**Affiliations:** 1 Kunming Edible Fungi Institute of All China Federation of Supply and Marketing Cooperatives, Kunming 650221, China; fengyunli1985@126.com (Y.F.); lsxcary@163.com (S.L.); jianying0818@163.com (J.L.); m13577016651@163.com (M.M.); ynjcyzf152@126.com (Z.Y.); m13508716295@163.com (L.S.); wykygx@163.com (X.G.); 2 Yunnan Academy of Edible Fungi Industry Development, Kunming 650221, China; fylavender@126.com

**Keywords:** *Tricholoma matsutake*, volatile flavor, GC×GC-TOF MS, Network analysis, fruiting bodies

## Abstract

Two-dimensional gas chromatography-time-of-flight mass spectrometry (GC × GC-TOF MS) was employed to analyze the volatile flavor compounds (VOCs) of *Tricholoma matsutake* samples from six different geographical regions: CX (Chuxiong), DL (Dali), DQ (Diqing), JL (Yanji), SC (Xiaojin) and XZ (Linzhi). The result indicate that a total of 2730 kinds of VOCs were identified from the fruiting bodies of six *T. matsutake* samples. The primary types of volatile organic compounds identified were 349 alcohols, 92 aldehydes, 146 carboxylic_acids, 311 esters, 742 organoheterocyclic compounds, 630 hydrocarbons, 381 ketones, 51 organic acids, and 28 derivatives and organosulfur compounds. Furthermore, PCA and PLS-DA analysis from the GC×GC-ToF-MS showed that samples from different regions could be distinguished by their VOCs. Network analysis revealed that 33 aroma compounds were identified as markers for distinguishing the samples from the six regions. The sensory attributes sweet, fruity, green, waxy, and floral were found to be more significant to the flavor profile of *T. matsutake*. 1-Nonanol, 2-Nonanone, Nonanoic acid, ethyl ester, 1-Undecanol, 2-Undecanone, Octanoic acid, ethyl ester, 2H-Pyran, and tetrahy-dro-4-methyl-2-(2-methyl-1-propenyl)- primarily contribute to the differences in the aroma characteristics among six *T. matsutake* samples. The results also provide a theoretical and practical foundation for the flavor compounds of these precious edible fungi in different regions.

## 1. Introduction

*Tricholoma matsutake* (S. Ito & S. Imai) Singer (*T. matsutake*) is a well-known wild edible mushroom, distributed in Asia, Europe, and North America [1]. In East Asia, specifically China, Japan and Korea, *T. matsutake* is renowned for its distinctive flavor, making it a sought-after ingredient in these culinary traditions. This mushroom has been found to contain high concentrations of proteins and amino acids. In addition, minerals, unsaturated fatty acids, vitamins, dietary fiber and a variety of other nutrients have been identified within it [2,3]. Furthermore, it also contains polysaccharides, polysaccharide–protein complex fractions and other bioactive components, which have demonstrated significant medicinal and nutritional value [4,5]. Despite its popularity, it is yet to be cultivated artificially, and all of the *T. matsutake* fresh fruiting bodies come from the wild.

The primary distribution regions of *T. matsutake* in China are Yunnan, Sichuan, Tibet and Jilin. Geographical isolation has been shown to be associated with genetic variations, differential nutritional value and divergent flavor characteristics [5,6]. Volatile flavor compounds have been used as chemical indicators for the identification and classification of specific species [6,7]. In the previous investigations, a total 66 VOCs were identified from Sichuan (Dechang, Daofu, Maerkang) by GC-MS [6]. Besides Sichuan, there are many other regions in China that have *T. matsutake*, such as Yunnan, Xizang, and Jilin. The evaluation of the volatile substances of *T. matsutake* from different regions is beneficial in understanding the influence of environmental conditions on *T. matsutake* and providing a scientific basis for the industrial production of *T. matsutake* [6].

Two-dimensional gas chromatography-time-of-flight mass spectrometry (GC×GC-TOF MS) is a novel technology that combines GC with high resolution and sensitivity, enabling the detection of a greater number of volatile organic compounds. Due to its strong peak separation ability, it has been widely applied in the research of various foods, especially in separating numerous subtle volatiles in complex sample matrices [8], such as coffee [9], Baijiu [10], cream [11], and Chinese dry-cured hams [12]. With a remarkable spectral acquisition rate reaching 500 spectra per second, this system demonstrates exceptional throughput for resolving thousands of chemical constituents in time-critical analyses. Previous studies on palm and palmist oils have shown that GC×GC-ToF-MS detects more VOCs, particularly fatty acid methyl esters, than GC-MS [13].

So far, there has been no research on the volatile flavor compounds of *T. matsutake* fruiting bodies in different regions of China. The composition and content of volatile compounds are crucial in determining the unique aromas of food, and serve as important indicators for evaluating quality. In this study, our research group conducted an analysis of the volatile compounds by using GC×GC-TOF MS on *T. matsutake* fruiting bodies samples from six different geographical regions: CX (Chuxiong), DL (Dali), DQ (Diqing), JL (Yanji), SC (Xiaojin) and XZ (Linzhi). The purpose is to find the volatile substances that can distinguish *T. matsutake* from these places, and to provide the basis for the industrialization of *T. matsutake*.

## 2. Materials and Methods

### 2.1. Fungal Materials and Sample Production

**Figure 1:**
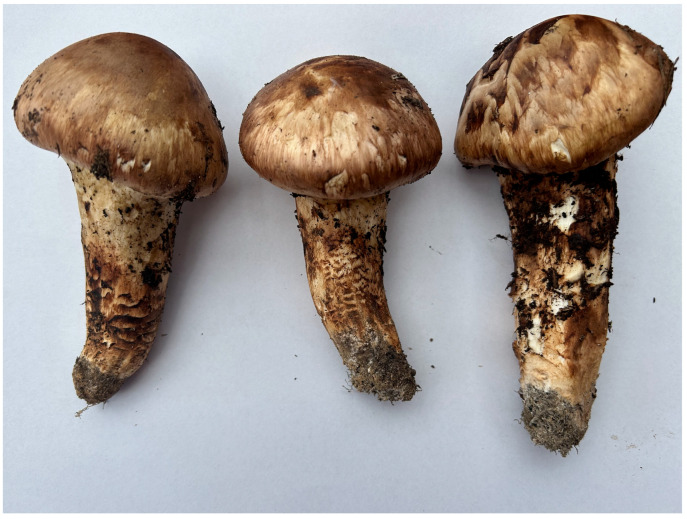
The fruiting bodies of *T. matsutake*.

The fruiting bodies of *T. matsutake* samples (Figure 1) were collected from six different geographical regions—Chuxiong (Yunnan province, China), Dali (Yunnan province, China), Diqing (Yunnan province, China), Yanji (Jilin province, China), Xiaojin (Sichuan province, China) and Linzhi (Xizang province, China)—in August of 2024, and were named CX, DL, DQ, JL, SC, and XZ, respectively. We collected three fruiting bodies from each region. The detailed information about these geographical regions is shown in Table 1. *T. matsutake* is selected based on uniform size, the absence of mechanical damage, and freedom from insect infestation. After removing the surface contaminants, *T. matsutake* samples were longitudinally sectioned into uniform slices of approximately 5 mm thickness and then stored in polyethylene cryotubes at a temperature of −80 °C for subsequent analyses. The experiments were performed over three replicates for each sample.

### 2.2. GC×GC-TOF MS Analysis

Analyses were performed using the LECO Pegasus^®^ 4D instrument (LECO, St. Joseph, MI, USA), which consists of an Agilent 8890A GC (Agilent Technologies, Palo Alto, CA, USA) equipped with a split/splitless injector, a two-stage cryogenic modulator (LECO), and a time-of-flight mass spectrometry (TOFMS) detector (LECO). We weighed 0.5 g of each sample of *T. matsutake* and placed them into a 20 mL headspace vial. The sample was then extracted using solid-phase micro-extraction (SPME). The SPME was incubated at 80 °C for 10 min, extracted for 25 min, and desorbed at the GC injection port for 5 min. Subsequently, GC×GC-TOF MS analysis was performed. The first-dimensional chromatographic column was DB-Heavy Wax (30 m × 250 μm × 0.5 μm), and the second-dimensional chromatographic column was Rxi-5Sil MS (2 m × 150 μm × 0.15 μm) (Restek, Bellefonte, PA, USA). The carrier gas was high-purity helium (>99.999%), and the flow rate was set at 1.0 mL/min. The oven temperature program was as follows: initially maintain the oven temperature at 50 °C for 2 min, then increase to 230 °C at a rate of 5 °C/min and hold for 5 min. The secondary oven temperature was set 5 °C higher than that of the primary oven. The modulator temperature was always maintained at 15 °C above that of the second column. The modulator was operated with a 6.0 s modulation period. The GC injector temperature was set 250 °C. The final results of the volatile compounds were expressed as normalized intensity, and are available in Table S1.

### 2.3. Statistical Analysis

The flavor compounds of the samples were identified using the NIST2020 database and Chroma TOF software (version 4.44). Following the execution of comprehensive data analysis employing Chroma TOF software, the following information was obtained: the nomenclature of each compound, the retention times, the CAS numbers, the RI information from the database, the actual RI calculated for normal alkanes C7-C30, and the peak areas of the samples. The final analysis result was derived by integrating these interpretive pieces of information. To facilitate the comparison of data across different scales, the original data underwent total peak area normalization or internal standard method normalization processing [14]. This project used internal standards for standardization. Principal component analysis (PCA) and partial least squares–discriminant analysis (PLS-DA) were performed using SIMCA-P (v13.0) and the R language ropls package [15]. All flavor compounds in nontargeted flavor omics and sensory annotation were obtained from the following databases: Odor database (Odor Thresholds for Chemicals with Established Health Standards, 2nd Edition) and Flavordb database (https://cosylab.iiitd.edu.in/flavordb/) (accessed on 18 December 2024).

## 3. Results

### 3.1. Statistical Identification of VOCs in Six T. matsutake Samples from Different Regions Using GC×GC-ToF MS

**Figure 2:**
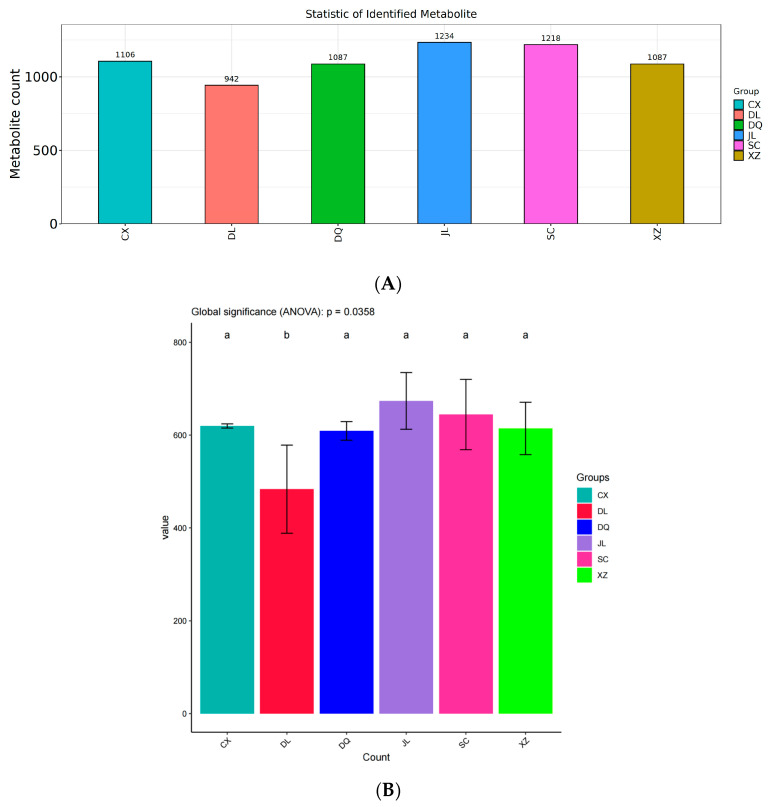
Statistic of identified VOCs of six *T. matsutake* samples from different regions using GC×GC-ToF MS. (**A**) Statistics of identified VOCs of six *T. matsutake* samples from different regions. (**B**) Single-indicator multiple-comparison bar chart of volatiles of six *T. matsutake* samples. a, *p* < 0.05; b, *p* < 0.01. CX (Chuxiong), DL (Dali), DQ (Diqing), JL (Yanji), SC (Xiaojin), XZ (Linzhi).

The VOCs of six *T. matsutake* samples from different regions are shown in Figure 2, Table S1. The identification of VOCs has revealed that over 900 VOCs were observed in each sample. The VOCs count in JL (Yanji) was the highest, at 1234, and that in DL (Dali) was the lowest at 942. Figure 2B indicates that there were significant differences among the six *T. matsutake* samples. These results indicate that there are differences in VOCs among six *T. matsutake* regions. This difference may be closely related to its unique geographical environment. Yanji is located in the central region of the Changbai Mountain, and due to its high-latitude location, the average annual temperature in the region is low, ranging between 3 and 5 °C. These low-temperature climatic conditions result in a comparatively longer growth cycle for *T. matsutake* in this region, which may have promoted the accumulation of secondary metabolites, thereby affecting types and relative contents of volatile compounds in *T. matsutake* [1,6].

### 3.2. Analysis of VOCs Obtained by GC×GC-ToF MS

**Figure 3:**
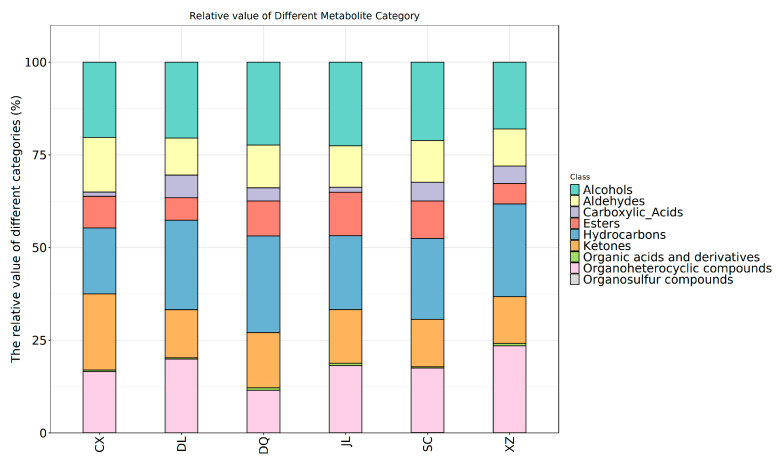
The relative contents of nine classes of VOCs in the six *T. matsutake* samples from different regions. CX (Chuxiong), DL (Dali), DQ (Diqing), JL (Yanji), SC (Xiaojin), XZ (Linzhi).

The detailed information on the VOCs identified by GC×GC-ToF MS is presented in Figure 3, Table S1. The six *T. matsutake* samples contained 2730 VOCs, including nine classes (Figure 3), i.e., alcohols (349), aldehydes (92), carboxylic_acids (146), esters (311), organoheterocyclic compounds (742), hydrocarbons (630), ketones (381), organic acids and derivatives (51) and organosulfur compounds (28).

As far as alcohols are concerned, JL (Yanji) and XZ (Linzhi) had the highest alcohol contents, both at 151, as indicated in Figure 3, Table S1. These were followed by SC (Xiaojin) (145), CX (Chuxiong) (144), DQ (Diqing) (137), and DL (Dali) (131). Alcohols can be divided into saturated alcohol and unsaturated alcohol; the saturated alcohol threshold is high, while the unsaturated alcohol threshold is relatively low, and it has unique odor, so it contributes more to the flavor [16,17,18]. The key alcohols of fresh *T. matsutake* were 3-Octanol, (E)-2-Octen-1-ol, 1-Octen-3-ol, 1-Octanol, 1-Hexanol, 1-Butanol and 3-methyl- [19,20]. 1-Octen-3-ol was present at relatively high levels in all *T. matsutake* samples (Table S1), with the highest relative content in the DL (Dali) sample at 117.42 and the lowest in the XZ (Linzhi) sample at 30.09, compared to other *T. matsutake* samples, which ranged from 50.41 to 62.19. 1-Octen-3-ol is known as mushroom alcohol and it has a strong, aromatic odor; decreases in concentrations will influence the odor characteristics of *T. matsutake* [6]. 1-Octen-3-ol is the product of linoleic acid in edible fungi, produced through autooxidation or enzymatic oxidation and cleavage [1,21,22]. 3-Octanol has a mushroom and moss aroma, and 1-Octanol has a chemical and sweet flavor [3].

The group of aldehydes consisted of a total of 92 VOCs (Table S1), which are found in SC (Xiaojin) (64), CX (Chuxiong) (60), JL (Yanji) (59), XZ (Linzhi) (58), DQ (Diqing) (55) and DL (Dali) (46). Aldehydes are known for their floral and fruity odor [23]. Acetaldehyde, Heptanal, Hexanal, Nonanal, Octanal, Pentanal, Butanal, 2-methyl-, Butanal, 3-methyl-, propanal and 2-methyl- were the main VOCs in all *T. matsutake* samples. Aldehydes are volatile flavor substances with a high content and relatively low odor threshold in edible fungi, which often significantly influence the characteristic flavor of the overall volatile profile [24,25]. They are the primary degradation products of lipid oxidation, although some can also be produced through Maillard-induced amino acids degradation [3,26]. Butanal, 3-methyl-, a branched Strecker aldehyde, was abundant in all *T. matsutake* samples, and it has a chocolate aroma. Hexanal has a fresh grass aroma, propanal, 2-methyl- has grassy and spicy aroma, Butanal, 2-methyl- has a coffee and nut aroma, Pentanal has a fermented bread and nut aroma, and Heptanal has a wine flavor [23,27].

Carboxylic acids originate from the hydrolysis of fats into short-chain volatile fatty acids, or the degradation of amino acids [28]. Table S1 shows that the major carboxylic acids were acetamide, N-2-propenyl-, propanoic acid, 2-methyl-, propanoic acid, 2,2-dimethyl-, anhydride with diethylborinic acid, Acetic acid, [(aminocarbonyl)amino]oxo-, and 1-Methylcyclopropanecarboxylic acid. They were the most abundant in DQ (Diqing) (66), and least present in DL (Dali) (38).

Esters usually add fruity and floral flavors to mushrooms, and they are generally produced by non-enzymatic catalyzed reactions of alcohols and organic acids or enzyme-catalyzed reactions involving microorganisms [29]. In six *T. matsutake* samples, 2-Propenoic acid, butyl ester, 2-Propenoic acid, ethyl ester, acetic acid, octyl ester, Benzenepropanoic acid, ethyl ester, Benzenepropanoic acid, methyl ester, Butanoic acid, 1-ethenylhexyl ester, Butanoic acid, ethyl ester, Decanoic acid, ethyl ester, Dodecanoic acid, ethyl ester, Dodecanoic acid, methyl ester, Ethyl Acetate, Ethyl formate, Formic acid, octyl ester, Heptanoic acid, ethyl ester, Heptanoic acid, methyl ester, Hexadecanoic acid, ethyl ester, Nonanoic acid, ethyl ester, Pentadecanoic acid, ethyl ester, Tetradecanoic acid and ethyl ester were the common representatives. The most abundant ester in six *T. matsutake* samples (Table S1) was Hexadecanoic acid, ethyl ester, which contributes a waxy, fruity and creamy aroma. It was particularly highly prevalent in CX (Chuxiong), at 55.34, and least in XZ (Linzhi), at 1.78.

Organoheterocyclic compounds were the predominant VOCs in six *T. matsutake* samples, including furans, furanones, pyrazine, pyranones and so on. They were the most abundant in JL (Yanji) (332) and the lowest in DL (Dali) (244). Pyrazine is a specific product of the Maillard reaction, and has a unique fragrance [30]; it is present as Pyrazine, Pyrazine, 2,5-dimethyl-, Pyrazine, ethyl-, and Pyrazine, methyl-. The most abundant furans in *T. matsutake* samples was 2-n-Butyl furan, which has a light fruit aroma, with sweet wine flavor.

Alkanes in hydrocarbons are primarily derived from the homolytic cleavage of the alkoxy radical of fatty acids, with usually a high threshold and no obvious flavor characteristics [31]. They do not significantly contribute to the flavor of *T. matsutake*, but they can complement each other with other aromatic substances to give *T. matsutake* # different flavors. Studies indicate that alkanes and alcohols can be converted into each other and change the sensory properties of foods, after which they can play a role in harmonizing and complementing the flavor of *T. matsutake* [19]. In our study, the amount of SC (Xiaojin) (281) was the highest, followed by JL (Yanji) (277), and DQ (Diqing) (229) was the lowest. Among them, the hydrocarbons with higher contents were Tetradecane, Pentadecane, Hexadecane, Heptadecane, Octadecane, Cyclohexane-d12, 1,3-Octadiene, and 2-Hexene, 3,5,5-trimethyl-.

Ketones are primarily produced by the oxidation of unsaturated lipids and Maillard reactions, and their thresholds are higher than those of aldehydes, among which short-chain ketones contribute fatty and burnt aroma notes, whereas their long-chain ketones contribute floral notes [27]. It is evident that the majority of ketones are characterized by relatively low odor thresholds, consequently exerting a more pronounced impact on the overall flavor profile [32]. The major ketones in the six *T. matsutake* samples were Acetone, 1-Octen-3-one, 2,3-Hexanedione, 2-Decanone, 2-Heptanone, 3-Heptanone, 2-Octanone, 3-Octanone, 2-Pentadecanone and 2-Undecanone. 1-Octen-3-one was the primary mushroom flavor substance found in fresh *T. matsutake*. It was particularly high in CX (Chuxiong) at 76.14, and lowest in XZ (Linzhi) at 0.04. 3-Octanone also contributes to the mushroom and butter taste of *T. matsutake* [19]. 2-Undecanone is characterized as a typical compound for fruity and green flavors, and is commonly associated with the flavor profiles of certain foodstuffs. Acetone has apple and pear flavors [23].

The formation of acids may occur as secondary reaction products of the thermal decomposition and thermal degradation of unsaturated fatty acids during drying [33]. In the six *T. matsutake* samples, there were only 51 organic acids and derivatives; the largest amount was in XZ (Linzhi) (26), followed by JL (Yanji) (23), DQ (Diqing) (21) and DQ (Diqing) (18), and CX (Chuxiong) (17) contained the least. The major organic acids and derivatives were Butanoic acid, 2-oxo-, Ethyl acetoacetate and Propanoic acid, 2-oxo-.

Organosulfur compounds are usually present in the volatile flavors of edible mushrooms. They often typically emit a strong, pungent odor [34]. It is worth noting that Dimethyl disulfide and Dimethyl trisulfide are the predominant flavor compounds of *Lentinula edodes* [35]. The presence of distinct odor compounds in various types of edible mushrooms gives rise to significantly different aroma characteristics. Table S1 shows that 28 organosulfur compounds were detected across the six *T. matsutake* samples. CX (Chuxiong) was found to have the highest abundance of organosulfur compounds (13), followed by SC (Xiaojin) (12), while the lowest was DL (Dali) (7). All *T. matsutake* samples contained Disulfide, dimethyl, which was confirmed to contribute to the characteristic flavor of *T. matsutake*.

### 3.3. PCA and PLS-DA Results of Six T. matsutake Samples in Different Regions

**Figure 4:**
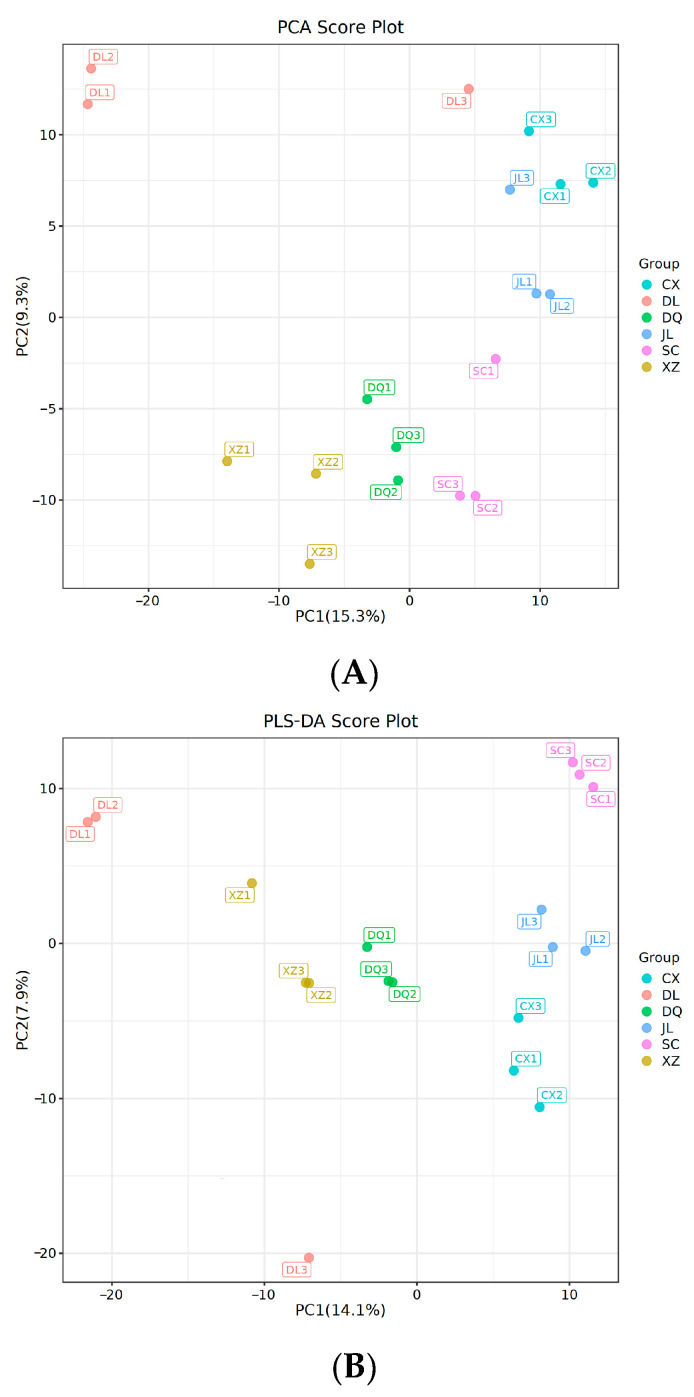
Volatile organic compounds detected in six *T. matsutake* samples from different regions. (**A**) PCA of volatiles of six *T. matsutake* samples; (**B**) PLS-DA of volatiles of six *T. matsutake* samples. CX (Chuxiong), DL (Dali), DQ (Diqing), JL (Yanji), SC (Xiaojin), XZ (Linzhi).

The PCA results of volatiles detected in the present study are shown in Figure 4A. In total, 888 volatile compounds were selected for analysis in the experiment. The PCA score plot distinctly shows the significant regional differentiation among the six *T. matsutake* samples based on their volatile flavor compounds. PCA is a widely utilized multivariate statistical analysis tool in the field of sample variance analysis. The primary advantage of PCA is its ability to simplify data and elucidate the interrelationships among different samples [36]. In our study, the contribution rates of PC1 and PC2 were 15.3% and 9.3%. The total contribution rates of PC1 and PC2 reached 24.6%. The distribution diagram indicates that six *T. matsutake* samples could be distinguished by PC1 and PC2, revealing distinct flavor relationships among the selected samples. Figure 4A indicates that two *T. matsutake* samples (DQ (Diqing), XZ (Linzhi)) were located close to each other. This may be because the geographical locations of the two places are relatively close, and the similarities of their environmental conditions, including climate and soil, lead to the formation of similar flavors. The geographical location and altitude differences of different producing areas resulted in a variety of climates and ecological environments, as a result of which *T. matsutake* presented different flavor characteristics.

Following the identification of distinct clustering patterns among the six *T. matsutake* samples through PCA, a systematic investigation was subsequently performed using PLS-DA to elucidate the key variables responsible for the observed inter-sample differentiations. As shown in Figure 4B, samples from different regions can be effectively distinguished using PLS-DA.

### 3.4. Different Flavor Substances of Six T. matsutake Samples in Different Regions

**Figure 5:**
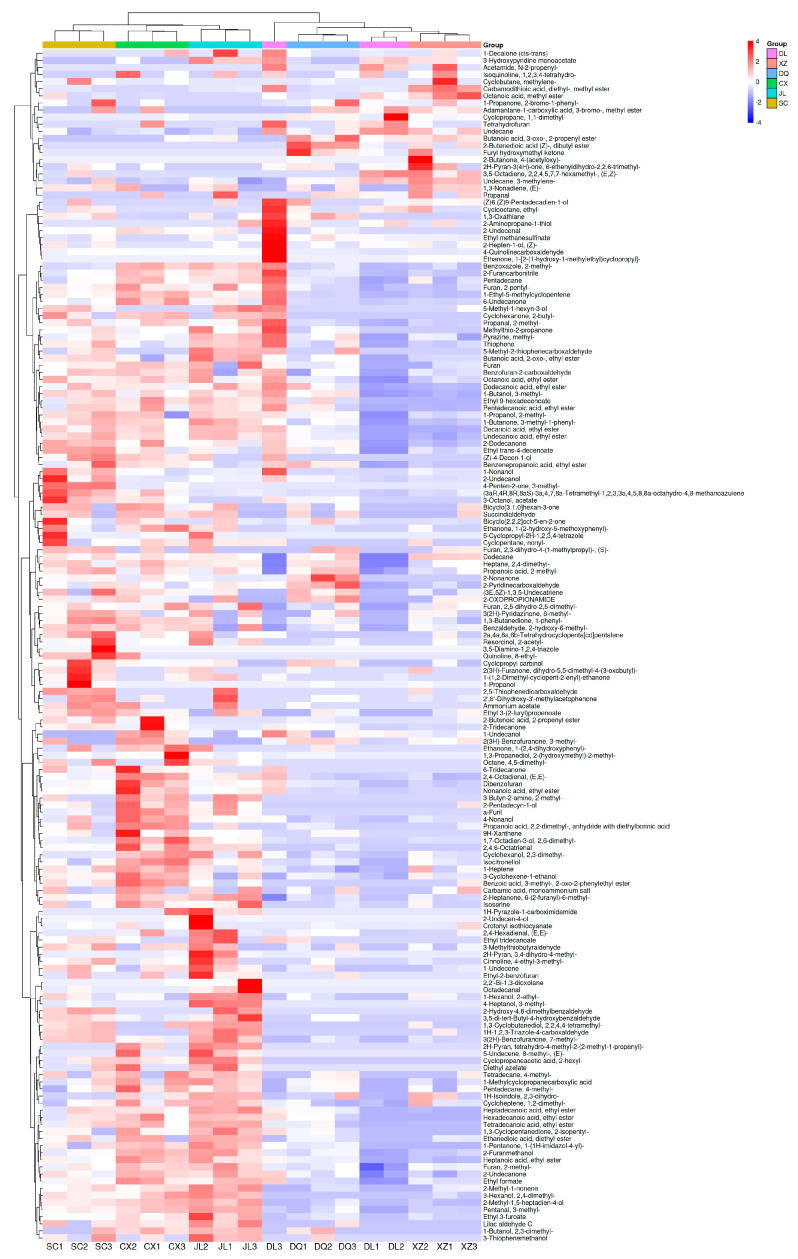
Cluster heatmap of volatile flavor compounds of six *T. matsutake* samples in different regions. CX (Chuxiong), DL (Dali), DQ (Diqing), JL (Yanji), SC (Xiaojin), XZ (Linzhi).

To analyze the data obtained from GC×GC-TOF MS, a nonparametric test (specifically, one-way ANOVA) was utilized to analyze the identified 888 flavor compounds. The findings indicate that 168 flavor compounds exhibited significant variations among the six *T. matsutake* samples, suggesting their potential as crucial markers for differentiating regional samples (Figure 5). The relative contents are shown by the different colors in the figure. The more red the color, the higher the relative content, and the more blue, the lower the relative content. These markers included 26 alcohols, 15 aldehydes, 10 carboxylic_acids, 24 esters, 39 organoheterocyclic compounds, 24 hydrocarbons, 25 ketones, 3 organic acids and derivatives and 2 organosulfur compounds. VOCs of the six *T. matsutake* samples from different regions exhibited both common peaks and characteristic peaks, indicating that the compositions and contents of *T. matsutake* flavor compounds from different regions were both similar and different. By systematically analyzing the heatmap, we can further investigate the influences of geographical variations on the flavor compounds of *T. matsutake*, thus providing a scientific basis for subsequent research (such as production area tracing and quality regulation).

The samples of DL (Dali), DQ (Diqing) and XZ (Linzhi) were clustered together, which may be related to their similar geographical environments. There were significant differences in flavor compounds between CX (Chuxiong), JL (Yanji) and SC (Xiaojin). Compared with the samples from DL (Dali), DQ (Diqing) and XZ (Linzhi), in the SC (Xiaojin) sample, 2-Undecano, 4-Penten-2-one, 3-methyl-, 3-Octanol, acetate, Bicyclo[2.2.2]oct-5-en-2-one, Ethanone, (3aR,4R,8R,8aS)-3a,4,7,8a-Tetramethyl-1,2,3,3a,4,5,8,8a-octahydro-4,8-methanoazulene, 1-(2-hydroxy-5-methoxyphenyl)-, 5-Cyclopropyl-2H-1,2,3,4-tetrazole, Cyclopentane, nonyl-, 2a,4a,6a,6b-Tetrahydrocyclopenta[cd]pentalene, Resorcinol, 2-acetyl-, 3,5-Diamino-1,2,4-triazole, Quinoline, 8-ethyl-, Cyclopropyl carbinol, 2(3H)-Furanone, dihydro-5,5-dimethyl-4-(3-oxobutyl)-, 1-(1,2-Dimethyl-cyclopent-2-enyl)-ethanone1-, 1-Propanol were higher. 2-Butenoic acid, 2-propenyl ester, 2-Tridecanone, 1-Undecanol, Ethanone, 1-(2,4-dihydroxyphenyl)-, 1,3-Propanediol, 2-(hydroxymethyl)-2-methyl-, Octane, 4,5-dimethyl-, 6-Tridecanone, 2,4-Octadienal, (E,E)-, Dibenzofuran, Nonanoic acid, ethyl ester, 3-Butyn-2-amine, 2-methyl-, 2-Pentadecyn-1-ol, a-Furil, 4-Nonanol, Propanoic acid, 2,2-dimethyl-, anhydride with diethylborinic acid, 9H-Xanthene, 1,7-Octadien-3-ol, 2,6-dimethyl-, 2,4,6-Octatrienal were higher in the CX (Chuxiong) sample. And 1H-Pyrazole-1-carboximidamide, 2-Undecen-4-ol, Crotonyl isothiocyanate, 2,4-Hexadienal, (E,E)-, Ethyl tridecanoate, 2H-Pyran, 3,4-dihydro-4-methyl-, Cinnoline, 4-ethyl-3-methyl-, 1-Undecene, Ethyl-2-benzofuran, 2,2′-Bi-1,3-dioxolane, Octadecanal, 1-Hexanol, 2-ethyl-, 4-Heptanol, 3-methyl-, 2-Hydroxy-4,6-dimethylbenzaldehyde, 3,5-di-tert-Butyl-4-hydroxybenzaldehyde, 1,3-Cyclobutanediol, 2,2,4,4-tetramethyl-, 1H-1,2,3-Triazole-4-carboxaldehyde and 3(2H)-Benzofuranone, 7-methyl- were higher in the JL (Yanji) sample. This result reveals the influence of geography and climate on the flavor of *T. matsutake*.

### 3.5. Analysis of Sensory Flavor Characteristics

**Figure 6:**
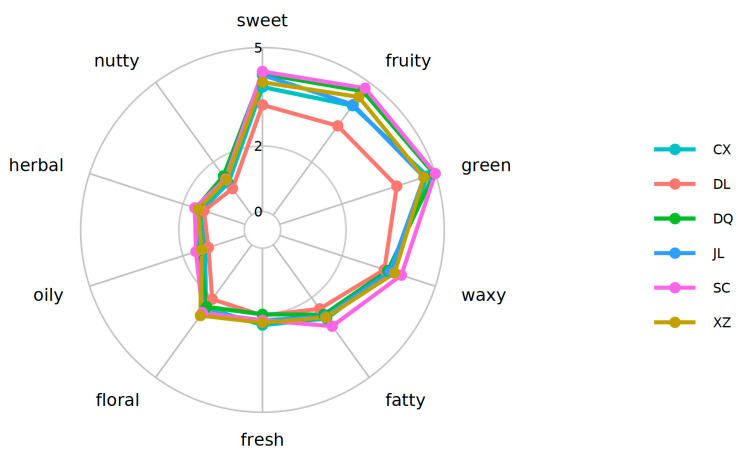
Radar map illustrating the sensory flavor characteristics of six *T. matsutake* samples from different regions. The outermost circle represents the sensory flavor characteristics, and the broken line indicates the frequency level of the corresponding flavor substance (the detection frequency is classified from 1 to 5, with the highest frequency being level 5). CX (Chuxiong), DL (Dali), DQ (Diqing), JL (Yanji), SC (Xiaojin), XZ (Linzhi).

The flavor profiles of products represent a complex composite of identifiable taste and odor features, as well as features that cannot be identified separately. In order to undertake a systematic analysis and comparison of flavor compounds, Flavordb [13] was employed as a sensory evaluation reference database. The possible flavor profiles of six *T. matsutake* samples are shown in Figure 6. In six *T. matsutake* samples, SC (Xiaojin) had the highest sweet, fruity, green, waxy and fatty flavors. The flavors of sweet, fruity, green, waxy, fatty and nutty were lowest in DL (Dali). The other flavors, such us fresh, floral, oily, herbal and nutty, were similar.

**Figure 7:**
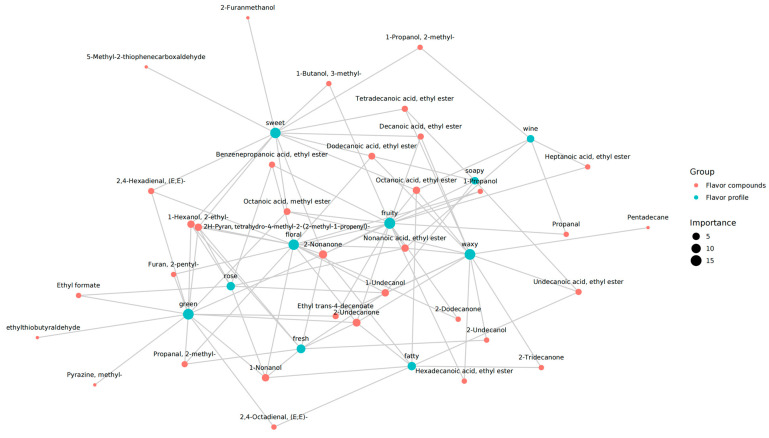
The correlation network between sensory attributes and aroma compounds (*p* < 0.05) of six *T. matsutake* samples from different regions. CX (Chuxiong), DL (Dali), DQ (Diqing), JL (Yanji), SC (Xiaojin), XZ (Linzhi).

Network analysis is regarded as a powerful tool for the investigation of potential correlations between flavor compounds and sensory attributes [37]. As demonstrated in Figure 7, volatiles have been shown to have a significant impact on flavor profiles, as evidenced by studies involving nontargeted flavoromics and online flavor databases. The green circle signifies the sensory feature, while the red circle represents the flavor compound. The larger the green circle, the greater the variety of flavor compounds associated with sensory characteristics, indicating its increased importance. The larger the red circle, the greater the number of sensory characteristics linked to the flavor compound, signifying the greater significance of the flavor substance. As indicated by the network, the most significant flavor compounds that have been identified as potentially key contributors to the aroma attributes of *T. matsutake* are further emphasized. The results show that 33 flavor compounds had a significant correlation with ten sensory attributes, suggesting that these 33 flavor compounds are key contributors to the flavor variations among six *T. matsutake* samples from different regions. Among them, the sensory attributes sweet, fruity, green, waxy, fatty, fresh, floral, rose, wine, and soapy were significantly correlated with 13, 16, 14, 13, 7, 8, 14, 7, 5, and 5 kinds of flavor compounds, respectively (Figure 7). So the sensory attributes sweet, fruity, green, waxy, and floral were more important. In the network, there is an interplay between sensory attributes and flavor compoundsl for example, 1-Nonanol, 2-Nonanone, Nonanoic acid, ethyl ester, 1-Undecanol, 2-Undecanone, Octanoic acid, ethyl ester, 2H-Pyran and tetrahydro-4-methyl-2-(2-methyl-1-propenyl)- were associated with *T. matsutake* aroma notes. In summary, these flavor compounds are primary contributors to the differences in the aroma characteristics among six *T. matsutake* samples.

## 4. Conclusions

In this study, GC×GC-ToF MS was utilized to analyze the volatile flavor substances present in six *T. matsutake* samples. The total number of VOCs identified in this study was found to be 2730. The sample from JL (Yanji) exhibited the highest count of VOCs, at 1234, whereas the sample from DL (Dali) had the lowest count, at 942. The primary types of volatile organic compounds were identified for alcohols, aldehydes, carboxylic acids, esters, organoheterocyclic compounds, hydrocarbons, ketones, organic acids and derivatives, and organosulfur compounds. Furthermore, the PCA and PLS-DA analysis from the GC×GC-ToF MS indicates that samples from different regions could be distinguished based on their VOCs. The results show that the VOCs of DQ (Diqing) and XZ (Linzhi) are the closest, which may be because the geographical locations of the two regions are relatively close. Network analysis enabled the identification of 33 aroma compounds that function as markers to distinguish samples from six regions. The sensory attributes sweet, fruity, green, waxy, and floral were more important to the flavor of *T. matsutake*. These flavor compounds, 1-Nonanol, 2-Nonanone, Nonanoic acid, ethyl ester, 1-Undecanol, 2-Undecanone, Octanoic acid, ethyl ester, 2H-Pyran, and tetrahydro-4-methyl-2-(2-methyl-1-propenyl)-, are primarily responsible for the variations in the aroma characteristics of the six *T. matsutake* samples.

The information presented in this study offers significant new insights into the flavor profile of *T. matsutake* in China. The results can also provide a theoretical and practical foundation for the flavors of precious edible fungal in different regions. However, this study also has certain limitations. The number of samples is limited, involving only six regions’ samples, which may not fully reflect all the characteristics of the volatile flavor substances of *T. matsutake* in China. Future research can expand the sample range to include more regions and varieties of matsutake mushrooms so as to further enhance the representativeness and accuracy of the results.

## Figures and Tables

**Table 1:** *Tricholoma matsutake* sampling sites in China.

Name	Collection Places	Geographic Position
CX	Nanhua, Chuxiong, Yunnan province, China	N 24°51′29″, E 100°48′32″
DL	Jianchuan, Dali, Yunnan province, China	N 26°35′9″, E 99°40′9″
DQ	Shangri-La, Diqing, Yunnan province, China	N 27°54′14″, E 99°38′14″
JL	Yanji, Jilin province, China	N 42°51′1″, E 129°29′59″
SC	Xiaojin, Aba, Sichuan province, China	N 30°57′49″, E 102°17′58″
XZ	Linzhi, Xizang province, China	N 29°53′53″, E 93°26′42″

## Data Availability

The original contributions presented in the study are included in the article/Supplementary Material, further inquiries can be directed to the corresponding authors.

## Supplementary Materials

The following supporting information can be downloaded at: https://www.mdpi.com/article/10.3390/foods14101824/s1, Table S1: GC×GC-ToF MS detected VOCs of six *T. matsutake* samples from different regions.

## References

[1] Guo Y., Chen D., Dong Y., Ju H., Wu C., Lin S. (2018). Characteristic Volatiles Fingerprints and Changes of Volatile Compounds in Fresh and Dried *Tricholoma Matsutake* Singer by HS-GC-IMS and HS-SPME-GC-MS. J. Chromatogr. B Analyt Technol. Biomed. Life Sci..

[2] Roncero-Ramos I., Delgado-Andrade C. (2017). The Beneficial Role of Edible Mushrooms in Human Health. Curr. Opin. Food Sci..

[3] Li M., Du H., Lin S. (2021). Flavor Changes of *Tricholoma Matsutake* Singer under Different Processing Conditions by Using HS-GC-IMS. Foods.

[4] Hoshi H., Yagi Y., Iijima H., Matsunaga K., Ishihara Y., Yasuhara T. (2005). Isolation and Characterization of a Novel Immunomodulatory α-Glucan−Protein Complex from the Mycelium of *Tricholoma Matsutakein* Basidiomycetes. J. Agric. Food Chem..

[5] Ding X., Hou Y. (2012). Identification of Genetic Characterization and Volatile Compounds of *Tricholoma Matsutake* from Different Geographical Origins. Biochem. Syst. Ecol..

[6] Li Q., Zhang L., Li W., Li X., Huang W., Yang H., Zheng L. (2016). Chemical Compositions and Volatile Compounds of *Tricholoma Matsutake* from Different Geographical Areas at Different Stages of Maturity. Food Sci. Biotechnol..

[7] Ouzouni P.K., Koller W., Badeka A.V., Riganakos K.A. (2009). Volatile Compounds from the Fruiting Bodies of Three *Hygrophorus* Mushroom Species from Northern Greece. Int. J. Food Sci. Tech..

[8] Tranchida P.Q., Franchina F.A., Dugo P., Mondello L. (2014). Comprehensive Two-dimensional Gas Chromatography-mass Spectrometry: Recent Evolution and Current Trends. Mass. Spec. Rev..

[9] Fang X., Chen Y., Gao J., Run Z., Chen H., Shi R., Li Y., Zhang H., Liu Y. (2023). Application of GC–TOF/MS and GC×GC–TOF/MS to Discriminate Coffee Products in Three States (Bean, Powder, and Brew). Foods.

[10] Yu Y., Nie Y., Chen S., Xu Y. (2022). Characterization of the Dynamic Retronasal Aroma Perception and Oral Aroma Release of Baijiu by Progressive Profiling and an Intra-Oral SPME Combined with GC × GC-TOFMS Method. Food Chem..

[11] Schütt J., Schieberle P. (2017). Quantitation of Nine Lactones in Dairy Cream by Stable Isotope Dilution Assays Based on Novel Syntheses of Carbon-13-Labeled γ-Lactones and Deuterium-Labeled δ-Lactones in Combination with Comprehensive Two-Dimensional Gas Chromatography with Time-of-Flight Mass Spectrometry. J. Agric. Food Chem..

[12] Li W., Chen Y.P., Blank I., Li F., Li C., Liu Y. (2021). GC × GC-ToF-MS and GC-IMS Based Volatile Profile Characterization of the Chinese Dry-Cured Hams from Different Regions. Food Res. Int..

[13] Garg N., Sethupathy A., Tuwani R., Nk R., Dokania S., Iyer A., Gupta A., Agrawal S., Singh N., Shukla S. (2017). FlavorDB: A Database of Flavor Molecules. Nucl. Acids Res..

[14] Dunn W.B., Broadhurst D., Begley P., Zelena E., Francis-McIntyre S., Anderson N., Brown M., Knowles J.D., Halsall A., Haselden J.N. (2011). Procedures for Large-Scale Metabolic Profiling of Serum and Plasma Using Gas Chromatography and Liquid Chromatography Coupled to Mass Spectrometry. Nat. Protoc..

[15] Thévenot E.A., Roux A., Xu Y., Ezan E., Junot C. (2015). Analysis of the Human Adult Urinary Metabolome Variations with Age, Body Mass Index, and Gender by Implementing a Comprehensive Workflow for Univariate and OPLS Statistical Analyses. J. Proteome Res..

[16] Hiraide M., Miyazaki Y., Shibata Y. (2004). The Smell and Odorous Components of Dried Shiitake Mushroom, *Lentinula Edodes* I: Relationship between Sensory Evaluations and Amounts of Odorous Components. J. Wood Sci..

[17] Zhang J., Cao J., Pei Z., Wei P., Xiang D., Cao X., Shen X., Li C. (2019). Volatile Flavour Components and the Mechanisms Underlying Their Production in Golden Pompano (*Trachinotus Blochii*) Fillets Subjected to Different Drying Methods: A Comparative Study Using an Electronic Nose, an Electronic Tongue and SDE-GC-MS. Food Res. Int..

[18] Bao C., Guan C., Xin M., Teng X., Liu T., Wang D. (2022). Effect of Roasting on Volatile Flavor Compounds of *Stropharia rugoso-annulata* Analyzed by Headspace-Solid Phase Microextraction-Gas Chromatography-Mass Spectrometry Combined with Electronic Nose. Food Sci..

[19] Cho I.H., Kim S.Y., Choi H.-K., Kim Y.-S. (2006). Characterization of Aroma-Active Compounds in Raw and Cooked Pine-Mushrooms (*Tricholoma Matsutake* Sing.). J. Agric. Food Chem..

[20] Pueschel V.A., Schieberle P. (2020). Changes in the Key Aroma Compounds of Matsutake Mushroom (*Tricholoma Matsutake* Sing.) from Canada during Pan-Frying Elucidated by Application of the Sensomics Approach. Eur. Food Res. Technol..

[21] Wurzenberger M., Grosch W. (1984). The Formation of 1-Octen-3-Ol from the 10-Hydroperoxide Isomer of Linoleic Acid by a Hydroperoxide Lyase in Mushrooms (*Psalliota Bispora*). Biochim. Biophys. Acta (BBA)-Lipids Lipid Metab..

[22] Combet E., Eastwood D.C., Burton K.S., Henderson J. (2006). Eight-Carbon Volatiles in Mushrooms and Fungi: Properties, Analysis, and Biosynthesis. Mycoscience.

[23] Dong H., Lu H., Jiang N., Li Q., Li Y., Shang X., Li Z., Wang Z., Song C., Zhou F. (2023). GC-IMS analyses of volatile organic compounds in *Lentinula edodes* at different ages. Mycosystema.

[24] Hou H., Liu C., Lu X., Fang D., Hu Q., Zhang Y., Zhao L. (2021). Characterization of Flavor Frame in Shiitake Mushrooms (*Lentinula Edodes*) Detected by HS-GC-IMS Coupled with Electronic Tongue and Sensory Analysis: Influence of Drying Techniques. LWT.

[25] Guo Q., Adelina N.M., Hu J., Zhang L., Zhao Y. (2022). Comparative Analysis of Volatile Profiles in Four Pine-Mushrooms Using HS-SPME/GC-MS and E-Nose. Food Control.

[26] Lai P., Li L., Wei Y., Sun J., Tang B., Yang Y., Chen J., Wu L. (2024). GC-IMS-Based Volatile Characteristic Analysis of *Hypsizygus Marmoreus* Dried by Different Methods. Foods.

[27] Zhang Q., Xu Y., Dong Q., Shu X., Zhang S., Xie L., Peng W., Wang H. (2025). Analysis and evaluation of volatile compounds in different fruiting bodies of different strains of *Morchella*. Mycosystema.

[28] Zhang Y., Gao P., Zhang W., Zhu H., Wang C., Xie N., Wang Y., Pang X., Marie-Laure F., Lü J. (2023). Free Fatty Acid Hydrolyzed with Lipases and Their Effects on Enzyme-Modified Cheese Flavor. Food Sci. Anim. Prod..

[29] Yang H., Li W., Zi L., Xu N., Guo Z., Chen B., Hua Y., Guo L. (2024). Comprehensive Analysis of the Dynamic Changes of Volatile and Non-Volatile Metabolites in *Boletus Edulis* during Processing by HS-SPME-GC-MS and UPLC-MS/MS Analysis. Food Chem. X.

[30] Liao Y., Ding Y., Wu Y., Du Q., Xia J., Jia J., Lin H., Benjakul S., Zhang B., Hu Y. (2023). Analysis of Volatile Compounds and Flavor Fingerprint in Hairtail (*Trichiurus Lepturus*) during Air-Drying Using Headspace-Gas Chromatography-Ion Mobility Spectrometry (HS-GC-IMS). Front. Nutr..

[31] Yang L., Niu L., Yu W., Deng L., Ye L., Wu X., He X., Fan Y. (2023). Analyses of volatile components of *Armillaria* spp. in the Changbai Mountain, Northeast China. Mycosystema.

[32] Li W., Zi L., Xu N., Yang H., Dong S., Qin F., Guo L. (2024). Identification of Characteristic Flavor Compounds of *Boletus Edulis* from Different Regions Based on by E-Nose, HS-GC-IMS and HS-SPME-GC-MS. Food Chem. X.

[33] Ma Y., Yu H., Huang F., Qiang Y., Han D., Jia Q., Zhang C. (2023). Analysis of differences in nutritional quality and volatile flavor components of daylily from different regions. China Condiment.

[34] Huang L., He C., Si C., Shi H., Duan J. (2023). Nutritional, Bioactive, and Flavor Components of Giant Stropharia (*Stropharia Rugoso-Annulata*): A Review. JoF.

[35] Liu Q., Bau T., Jin R., Cui X., Zhang Y., Kong W. (2022). Comparison of Different Drying Techniques for Shiitake Mushroom (*Lentinus Edodes*): Changes in Volatile Compounds, Taste Properties, and Texture Qualities. LWT.

[36] Pantoja-Benavides A.D., Garces-Varon G., Restrepo-Díaz H. (2021). Foliar Growth Regulator Sprays Induced Tolerance to Combined Heat Stress by Enhancing Physiological and Biochemical Responses in Rice. Front. Plant Sci..

[37] He Y., Liu Z., Qian M., Yu X., Xu Y., Chen S. (2020). Unraveling the Chemosensory Characteristics of Strong-Aroma Type Baijiu from Different Regions Using Comprehensive Two-Dimensional Gas Chromatography-Time-of-Flight Mass Spectrometry and Descriptive Sensory Analysis. Food Chem..

